# Smartphone-Based Care Platform Versus Traditional Care in Primary Knee Arthroplasty in the United States: Cost Analysis

**DOI:** 10.2196/46047

**Published:** 2025-02-03

**Authors:** Jess H Lonner, Ashwini Naidu-Helm, David Van Andel, Mike B Anderson, Richard Ditto, Roberta E Redfern, Jared Foran

**Affiliations:** 1 Rothman Orthopaedics, Rothman Orthopaedic Institute, 825 Old Lancaster Rd, Suite 100, 140, Philadelphia, PA, 19010, United States, 1 8003219999; 2 Health Economic and Market Access, Zimmer Biomet, Warsaw, IN, United States; 3 Clinical Affairs, Zimmer Biomet, Warsaw, IN, United States; 4 Panorama Orthopedics and Spine Center, Golden, CO, United States

**Keywords:** telerehabilitation, telehealth, telemedicine, rehabilitation, physiotherapy, mobile health, knee arthroplasty

## Abstract

Cost savings were achieved with the use of a smartphone-based care management platform, considering several health care resources following knee arthroplasty procedures without negatively impacting clinical outcomes.

## Introduction

The number of knee arthroplasty procedures performed in the United States has increased annually, accompanied by rises in health care resource use and costs [1]. Risk-sharing and bundled payment plans have been implemented with the goal of improving care and controlling costs through the sharing of financial responsibility for a 90-day joint replacement episode-of-care (EOC) [2]. Approximately 36% of the EOC cost has been attributed to postdischarge services [3]. In-person outpatient physiotherapy (PT) has been the standard of care (SoC) postoperatively but is associated with significant cost and may be overused following uncomplicated knee arthroplasty [4]. Telerehabilitation and mobile health delivery of PT has gained popularity in recent years [5]. The aim of this study was to compare costs of a smartphone-based care management platform (sbCMP) with traditional care in adult patients undergoing knee arthroplasty.

## Methods

### Study Design and Analysis

Patients undergoing primary total or partial knee arthroplasty were randomized (block randomization, block size of 4) to receive institutional SoC (control group) or sbCMP (treatment group) using the mymobility app (Zimmer Biomet), as previously described [6]. The 90-day health care intervention collected information on PT visits, manipulations under anesthesia, non-SoC physician visits, all-cause readmissions, emergency department (ED) visits, and urgent care visits.

Costs associated with the use of each resource were estimated from multiple sources [4,7,8]. The cost of the sbCMP was estimated based on the maximum cost per patient. Costs were multiplied across number of uses; the average cost per patient was calculated using the entire cohort as the denominator and compared between groups by a 2-tailed student *t* test. Costs in the noncrossover treatment group (patients who did not receive traditional PT in the original study) were also calculated. One-way and two-way deterministic sensitivity analyses were performed.

### Ethical Considerations

The multicenter randomized controlled trial (ClinicalTrials.gov NCT03737149) received central Institutional Review Board approval (20182103). All patients voluntarily provided written informed consent upon enrollment, with the opportunity to withdraw at any time. Smartwatches were provided (approximately US $329 in value). Data were deidentified prior to analyses.

## Results

Preoperative patient characteristics as well as baseline and postsurgical clinical outcomes were similar between the two groups [6]. The estimated costs associated with health care resources are presented in Table 1. The number of events was lower in the treatment group in each category except for non-SoC physician office visits. Patients using the sbCMP had significantly fewer ED visits and readmissions. The average cost per patient was approximately US $732 lower in the treatment group after including the cost of the sbCMP. The reduction in the number of in-person PT visits accounted for the bulk of the potential savings in the cohort, with costs approximately US $400 less in the treatment group. The average number of PT visits in the control and sbCMP groups were 9.75 (SD 3.98) and 5.40 (SD 5.51), respectively. Considering only noncrossovers, average costs were further reduced to approximately US $186 per patient through 90 days (Table 2). Sensitivity analyses demonstrated cost savings across all scenarios (Multimedia Appendix 1).

**Table 1.** Estimated costs associated with resource use as a function of treatment group.

Variable	Cost per unit (US $)^a^	Control arm (n=244)			Treatment arm (n=208)			*P* value
		Frequency, n	Total cost per group (US $)	Average cost per patient (US $)	Frequency, n	Total cost per group (US $)	Average cost per patient (US $)	
Physiotherapy^b^	126^c^ [4]	1736	218,736	896.46	799	100,674	484.01	<.001
Readmission	9723 [7]	16	155,568	637.57	5	48,615	233.73	.055
ED visit^d^	519 [8]	16	8304	34.03	5	2595	12.48	.03
MUA	1549	10	15,490	63.48	4	6196	29.79	.20
Physician office visit	27	67	1809	7.41	77	2079	10.00	.18
Urgent care visit	100	3	300	1.23	2	200	0.96	.79
Smartphone-based care platform	137	0	0	0.00	208	28,496	137.00	—
Total			400,207	1640.19		188,855	907.96	.001
Cost reduction			—	—		211,352	732.24	

a Reference citations indicated the studies from which the costs were derived.

b Estimates based on categorical collection of physiotherapy visits used. Categories included 1‐3 visits, 4‐6 visits, 7‐9 visits, 10‐12 visits, and 13 or more visits. Given the largest category did not include an upper bound, the lowest number of visits in each category was applied, providing conservative estimates in both groups.

c Weighted mean accounting for location (home, outpatient, or both).

d Represents only emergency department visits that did not result in admission. Readmissions associated with an emergency department visit are categorized only as readmission, without impacting emergency department visit rate.

e ED: emergency department.

f MUA: manipulations under anesthesia.

**Table 2.** Resource use and estimated costs within the treatment noncrossover group, where in-person physiotherapy was not used.

Variable	Treatment group noncrossover (n=61), n	Total cost per group (US $)	Average cost per patient (US $)
Physiotherapy	0	0	0
Readmission	0	0	0
ED^a^ visit	1	519	8.51
MUA^b^	1	1549	25.39
Physician office visit	34	918	15.05
Urgent care visit	0	0	0
Smartphone-based care platform	61	8357	137
Total		11,343	185.95

a ED: emergency department.

b MUA: manipulations under anesthesia.

## Discussion

This analysis demonstrated cost savings for patients using the sbCMP for self-directed rehabilitation following primary knee arthroplasty. Most savings were derived from the reduction in PT, the most common resource used. Cost savings were also achieved with the sbCMP for all health care resources except physician office visits. It is possible that this increase in office visits is the result of improved communication between patients and their care team via real-time messaging. This may have prompted additional unexpected office visits for postoperative concerns, which may have prevented more costly ED visits or readmissions. Alternately, this could be due to a patient need for feedback and assurance not received during in-person PT.

To our knowledge, this is the first cost comparison analysis of this care management platform compared with traditional care. There are few studies of other telerehabilitation programs compared with traditional care in this context. One study reported reduced outpatient PT costs with increased use of web-based PT [9] and another reported overall EOC cost savings with telerehabilitation and acknowledged improved outcomes, without attributing costs to the type of event [10].

A key limitation of this analysis is the uncertainty regarding the generalizability of the list price for the sbCMP used in the analysis. Additionally, the cost data may not be generalizable. Postacute care use was not accounted for given it is not expected to differ between treatment modalities.

In conclusion, the sbCMP has a potential for robust cost savings without negatively impacting postsurgical outcomes in patients undergoing knee arthroplasty.

## Supplementary material

10.2196/46047 Multimedia Appendix 1 One- and two-way sensitivity analyses.

## Abbreviations

ED: emergency department
EOC: episode-of-care
PT: physiotherapy
sbCMP: smartphone-based care management platform
SoC: standard of care

## References

[1] Sloan M, Premkumar A, Sheth NP (2018). Projected volume of primary total joint arthroplasty in the U.S., 2014 to 2030. J Bone Joint Surg Am.

[2] Comprehensive care for joint replacement model. Centers for Medicare & Medicaid Services.

[3] Bozic KJ, Ward L, Vail TP, Maze M (2014). Bundled payments in total joint arthroplasty: targeting opportunities for quality improvement and cost reduction. Clin Orthop Relat Res.

[4] Yayac M, Moltz R, Pivec R, Lonner JH, Courtney PM, Austin MS (2020). Formal physical therapy following total hip and knee arthroplasty incurs additional cost without improving outcomes. J Arthroplasty.

[5] Cottrell MA, Galea OA, O’Leary SP, Hill AJ, Russell TG (2017). Real-time telerehabilitation for the treatment of musculoskeletal conditions is effective and comparable to standard practice: a systematic review and meta-analysis. Clin Rehabil.

[6] Crawford DA, Duwelius PJ, Sneller MA (2021). 2021 Mark Coventry Award: use of a smartphone-based care platform after primary partial and total knee arthroplasty: a prospective randomized controlled trial. Bone Joint J.

[7] Phillips JLH, Rondon AJ, Vannello C, Fillingham YA, Austin MS, Courtney PM (2019). How much does a readmission cost the bundle following primary hip and knee arthroplasty?. J Arthroplasty.

[8] Sibia US, Mandelblatt AE, Callanan MA, MacDonald JH, King PJ (2017). Incidence, risk factors, and costs for hospital returns after total joint arthroplasties. J Arthroplasty.

[9] Zachwieja E, Theosmy EG, Yacovelli SJ, Beatty EW, McGrath ME, Lonner JH (2020). Web-based self-directed exercise program is cost-effective compared to formal physical therapy after primary total knee arthroplasty. J Arthroplasty.

[10] Prvu Bettger J, Green CL, Holmes DN (2020). Effects of virtual exercise rehabilitation in-home therapy compared with traditional care after total knee arthroplasty: VERITAS, a randomized controlled trial. J Bone Joint Surg Am.

