# Supplementary material for: Smartphone-Based Care Platform Versus Traditional Care in Primary Knee Arthroplasty in the United States: Cost Analysis
Source: JMIR Mhealth Uhealth. 2025 Feb 3;13:e46047. doi: 10.2196/46047 (PMC11809938; doi:10.2196/46047)
Supplement: Multimedia Appendix 1 [file mhealth-v13-e46047-s001.docx]

| **One-way Sensitivity Analysis (Parameters varied at 20%)** | | | | |
| --- | --- | --- | --- | --- |
| **Parameter** | **Cost difference - lower value** | **Model result** | **Cost difference - upper value** | **Model result** |
| Physiotherapy cost | $649.75 | Cost reduction with use of Smartphone-based care platform | $814.73 | Cost reduction with use of Smartphone-based care platform |
| Readmission cost | $651.47 |  | $813.01 |  |
| ED visit cost | $727.92 |  | $736.55 |  |
| MUA cost | $725.50 |  | $738.97 |  |
| Physician office visit cost | $732.75 |  | $731.72 |  |
| Urgent Care visit cost | $732.18 |  | $732.29 |  |
| Smartphone-based care platform cost | $759.64 |  | $704.84 |  |
| **Two-way Sensitivity Analysis** | | | | |
| **Parameters** | **Cost difference** | | **Model result** | |
| No. of Physiotherapy visits (20% lower limit for control group and 20% upper limit for treatment group | $195.64 | | Cost reduction with use of Smartphone-based care platform | |
| 20% Higher physiotherapy visits and 20% Higher readmissions in treatment arm | $328.09 | |  |  |

One- and two-way sensitivity analyses.
